# Artificial Skin – Culturing of Different Skin Cell Lines for Generating an Artificial Skin Substitute on Cross-Weaved Spider Silk Fibres

**DOI:** 10.1371/journal.pone.0021833

**Published:** 2011-07-26

**Authors:** Hanna Wendt, Anja Hillmer, Kerstin Reimers, Joern W. Kuhbier, Franziska Schäfer-Nolte, Christina Allmeling, Cornelia Kasper, Peter M. Vogt

**Affiliations:** 1 Department of Plastic, Hand, and Reconstructive Surgery, Medical School Hannover, Hannover, Germany; 2 Institute of Technical Chemistry, Leibniz University of Hannover, Hannover, Germany; Institut Européen de Chimie et Biologie, France

## Abstract

**Background:**

In the field of Plastic Reconstructive Surgery the development of new innovative matrices for skin repair is in urgent need. The ideal biomaterial should promote attachment, proliferation and growth of cells. Additionally, it should degrade in an appropriate time period without releasing harmful substances, but not exert a pathological immune response. Spider dragline silk from *Nephila spp* meets these demands to a large extent.

**Methodology/Principal Findings:**

Native spider dragline silk, harvested directly out of *Nephila spp* spiders, was woven on steel frames. Constructs were sterilized and seeded with fibroblasts. After two weeks of cultivating single fibroblasts, keratinocytes were added to generate a bilayered skin model, consisting of dermis and epidermis equivalents. For the next three weeks, constructs in co-culture were lifted on an originally designed setup for air/liquid interface cultivation. After the culturing period, constructs were embedded in paraffin with an especially developed program for spidersilk to avoid supercontraction. Paraffin cross- sections were stained in Haematoxylin & Eosin (H&E) for microscopic analyses.

**Conclusion/Significance:**

Native spider dragline silk woven on steel frames provides a suitable matrix for 3 dimensional skin cell culturing. Both fibroblasts and keratinocytes cell lines adhere to the spider silk fibres and proliferate. Guided by the spider silk fibres, they sprout into the meshes and reach confluence in at most one week. A well-balanced, bilayered cocultivation in two continuously separated strata can be achieved by serum reduction, changing the medium conditions and the cultivation period at the air/liquid interphase. Therefore spider silk appears to be a promising biomaterial for the enhancement of skin regeneration.

## Introduction

The regeneration of skin poses an important challenge for plastic surgery. Skin is not only the biggest, but also one of the most complex organs with equally complex functions. Adult skin consists of 2 tissue layers: The keratinized, stratified epidermis, commonly consisting of keratinocytes that form the surface barrier layer; and the dermis, which is an underlying, vascularized, collagen-rich connective tissue that provides strength, resilience and nourishment of the epidermis. The predominant part of the dermis is the extracellular matrix, which is produced by fibroblasts. Appendages like sweat and sebaceous glands and hair cross both layers. Due to its glandular- and barrier- functions, skin protects from chemical and physical environmental effects and fulfils important tasks in the regulation of homeostasis [1]. Skin defects induce water, electrolytes and protein loss and make the invasion of bacteria possible. In consequence, damages to the integrity or the loss of large portions of the skin, e.g. caused by burns, may result in a significant disability or even signify a life-threatening situation. However, optimization in resuscitation, shock therapy, ventilation support and nutritional management of patients with major full-thickness burns and the comprehensive implementation of expert centres result in an improvement of survival rates [2] [3]. Today, early wound closure is one of the crucial points in burn treatment [4]. The “gold standard” for burn wound closure is the application of split-thickness autografts, harvested from uninjured areas. In severely burned patients with extensively injured areas, however, not enough donor sites are available to provide sufficient autografts.

Another challenge in therapeutical approaches for wound healing is represented by chronic wounds like pressure ulcers and leg ulcers. Due to the increasing average age of the population, many risk factors like immobilisation in long-term care settings and adult onset diabetes increase, too. As a consequence, chronic wounds have a high incidence and enormous medical and economic impact [5]. For example, in the USA 6.5 million people suffer from chronic wounds, occasioning estimated costs of 25 billion USD.[6]

In summary, increased survival rates of full-thickness burn patients, a high incidence of chronic wounds, and other extensive injuries result in a great demand for donor skin or artificial skin substitutes. It has been postulated that the ideal artificial skin substitute should be as similar as possible to the histological and the physiological function of native human skin. Most suitable would be an artificial skin substitute which is already colonized by at best autologous skin cells. It should possess mechanical stability, be biocompatible and readily adherent. Later, it should undergo controlled degradation at a comparable rate as the new tissue grows in without noxal environmental effects. Furthermore, it should be easy to handle and apply to the wound site [7]. Settlement of these claims depends in large parts on the (bio)material, which is used as scaffold for tissue engineering.

Matrices used routinely in therapeutic applications are made from naturally derived materials such as collagen (Apligraf) [8] [9] or from synthetic materials such as polylactide-co-glycolide polymers (Dermagraft) [10]. While promising data have been published, these materials have poor mechanical properties *in vivo* and there are concerns about disease transmission in natural materials. There are two key challenges: Upgrading the deficiencies in terms of low mechanical strength and degradation properties; and the fabrication of scaffolds, which have defined shapes and a complex porous internal architecture that can direct tissue growth [11]. At present, all of the engineered skin substitutes that are available for clinical use fail to fulfil the criteria for fully functional skin [12] [11].

Alternative materials for scaffolds are therefore being sought, and silks have been proposed as potential candidates. Despite centuries of use as sutures or wound coverage in order to stop haemorrhages and promote wound healing [13], silks have recently been rediscovered as useful biomaterials for many applications in clinical repair and as scaffolds for tissue engineering [14]. Spiders can produce a variety of silk in different silk glands for production of webs, such as orb webs, cocoons or dragline silk. Dragline silk, the spider's safety line, of *Nephila spp.* spiders has been studied extensively. It consists of 5 layers (from exterior to interior): a lipid coat for protection, a glycocoat to ensure water balance, a skin layer and the outer and inner core, consisting of major ampullate proteins [15]. The core proteins, called spidroin 1 and 2, form the main part of the silk fibre and it can be assumed that the mechanical properties of the fibre are based on their complex structural features[16].

There are numerous indications that silks might be proper material for tissue engineering. Spider silks display excellent mechanical features that even rival man-made, high-tech fibres: Spider dragline silk is 5 times tougher than Kevlar because it is more extendible [17] [18] and its strength of 1.1 GPa approaches that of steel [19]. Silks are stable at a large temperature range up to 250°C, flexible and insoluble in many organic and aqueous solvents as well as weak acids and bases [20] [21] but nevertheless, they seem to be slowly biodegradable [22], [23].

Another critical success factor for clinical use is biocompatibility. Spider silks do not have an immunogenic sericin coat like native silkworm silks and previous studies by Vollrath et al. showed that *Nephila spp*. dragline silk causes just a minor immunogenic response when implanted subcutaneously in pigs [24], [25]. In addition, it is thought that spider silks might also have bactericidal properties [26].

Furthermore, research has suggested that silk protein promotes cell attachment and growth as high as collagen and is ideal for the viability, growth and function of the cells [27] [28].

Many research groups focus on electrospinning with substantial progress. Because of the complex and not yet fully characterised construction of native silk, the design of artificial fibres with the same properties like native dragline silk proves to be still a complex challenge [29],[30] Recently, Xia et al., reported on the mechanical properties of a fibre that is comparable to native dragline silk. However, due to the outstanding characteristics of spider silks, the question arises if it might be possible to culture different types of skin cells in organotypic 3-dimensional structure on a matrix of native spider dragline silk. In this study, we constructed a simplified, bilayered skin equivalent containing fibroblasts and keratinocytes on woven native dragline silk.

## Results

### Frame design

**Figure 1:**
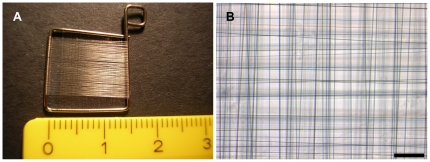
Frame design. Stainless steel straight wires with a diameter of 0,7 mm were bended to frames with side lengths of 1–1,5 cm and wound with spider dragline silk (A). Spider silk was woven in cross pattern to reach a mesh size between 10–100 µm (B). bar = 100 µm.

Native spider dragline silk could be woven on the self-made rectangle frames, resulting in regular meshworks with defined mesh sizes of 10-100 µm (Fig 1). The frames could be sterilized, were easy to handle with a cell culture forceps and cell culture compatible[27]. By winding spider silk around the frame of 0.7 mm thick steel wire, an upper and a lower mesh in the distance of the diameter of the steel wire were generated. In order to support cell migration through the interspace between upper and lower mesh, a clew of spider dragline silk was inserted between the meshes. Hereby, additional adhesion-points were given to MEF cells, which were now able to colonise the interspace along the spider silk fibres.

### Single culturing of fibroblasts or keratinocytes on spider silk frames

**Figure 2:**
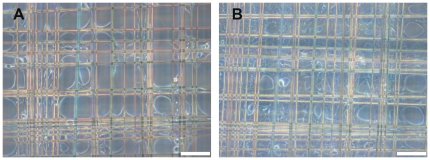
HaCaT cells, cultivated on spidersilk alone. 1^st^ (A) and 4^th^ (B) day after seeding. From the corners of the meshwork, cells spread into the meshes and reach confluence within 1 week. bar = 100 µm.

**Figure 3:**
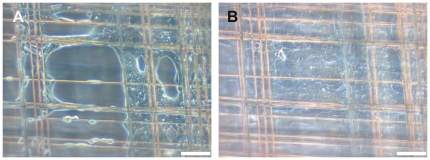
MEF cells, cultivated on spidersilk alone. 1^st^ (A) and 4^th^ (B) day after seeding. By comparison, MEF cells reach confluence earlier than HaCaT cells. bar = 100 µm.

**Figure 4:**
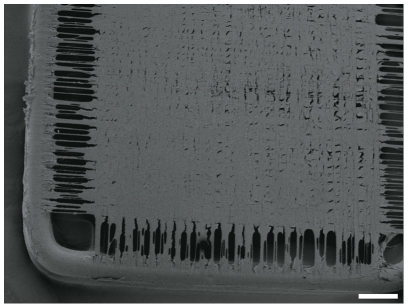
SEM of a confluent MEF cell layer on a frame with spider silk. The brink of the meshwork, where spider silk fibres do not cross each other is just rarely colonized, whereas a confluent cell-layer can be observed in the central meshwork. Culturing period took 5 days. bar = 1 mm.

MEF or HaCat cells could be cultured separately on the frames in standard cell culture media described above. Both cell types adhered to the spider silk and proliferated. Cells reached confluence by circular spreading into the interspace of the meshes. Compared to HaCat cells, MEF cells migrated and proliferated faster as concluded by their reaching confluence at an earlier time. Examination in daily light microscopy showed MEF cells reaching confluence on the frames within at most 5 days and HaCat cells within at most 7 days (Fig 2, 3 and 4). In live cell imaging, MEF cells showed especially fast migration and were able to cross the interspace of the meshes by using their cytoplasmatic extensions.

### Air-liquid interface culturing of frames

**Figure 5:**
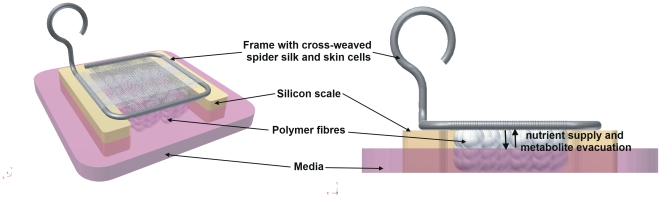
Cultivation at the air liquid interface. Frames with spider silk, seeded with MEF and HaCaT cells, are lifted on a silicone scale which is filled up with polymer fibres. The surface of the nutrient media is below the altitude of the frame. Media and frame are connected by the polymer fibres, which allow unilateral nutrient supply and metabolite evacuation by diffusion.

To achieve the bi-layered structure of a skin substitute, cells were added in two steps. The first cell type (MEF as dermis equivalent) was cultured under MEF-specific medium up to 14 days to generate a proper confluent cell layer. A special silicone scale was designed to lift up the frames for air/liquid interface cultivation in order to obtain epidermal organization of the subsequently added HaCaT cells. For nutrient supply and metabolite evacuation per diffusionem, polymer fibres were applied in the center of the scale. (Fig 5) PH alterations, due to metabolic activities were observed by indicators included in the used media on a daily basis. The used polymer fibres also prevented attachment of HaCaT cells on both sides of the frames.

### Assessment of long-term culture

After 35 days' culturing time, a confluent cell multilayer could be macroscopically observed. Light-microscopic observation showed that the cells had filled up the interspaces between spider silk fibres.

**Figure 6:**
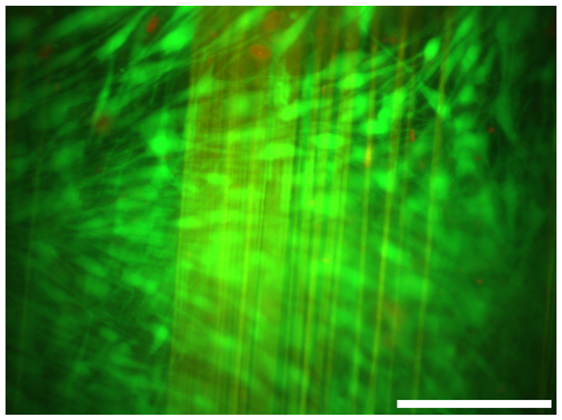
Life/Dead-Assay. Nearly 98% of the cells were detected by counting as being vital (green), dead cells (red) were just rarely observed. Spider silk fibres offered high autofluorescence.

Viability of MEF and HaCat cells in long term co-cultivation on spider silk frames was demonstrated with a LIVE/DEAD cell viability assay and fluorescence microscopy. After culturing time of 35 days, more than 98% of cells were detected as vital. Dead cells, identifiable by the emitted red fluorescent signal of ethidium bromide homodimer, were rarely observed. However, investigation was rendered difficult as spider silk fibres offered high autofluorescence (Fig.6).

### Histological analysis

**Figure 7:**
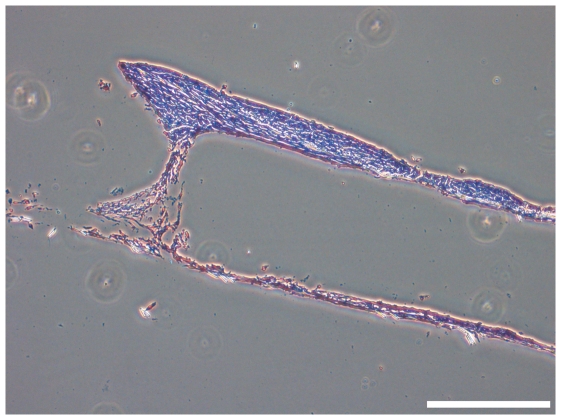
H&E stained section of a frame without a clew of spidersilk placed between upper and lower mesh. MEF cells were not able to bridge the 0.7 mm interspace in a cultivation period of 5 weeks. bar = 400 µm.

The histological overview of H & E stained paraffin cross-sections of frames without a clew of spider silk in the interspace between upper and lower mesh showed an empty interspace between the meshes. MEF cells were not able to bridge this 0.7 mm distance within 5 weeks of cultivation (Fig.7).

### Culturing of air-liquid interface frames in comparison to control frames

**Figure 8:**
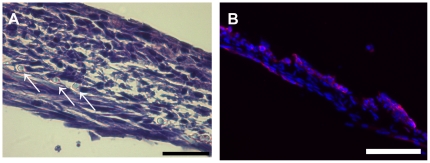
H&E stained section of a frame, which was cultured at the air liquid interphase without any special media supplements or serum reduction. Development of two continuously separated strata could not be achieved. Arrows mark cross sectioned spider silk fibres. (A) bar = 40 µm Immunofluorescence of the same frame, DAPI staining of all cells nuclei in blue, Involurin as specific marker for HaCaT cells in red (B).

Frames cultured at the air liquid interface in media without any special supplements (choleratoxin, hydrochortisone, A-2-P) or serum reduction showed just approximately an organtypic skin morphology (Fig.8). Both cell types showed, compared to the controls, increased proliferation and enhanced 3-dimensional alignment. Fibroblasts (MEF), as a dermal equivalent, showed long shaped morphology and keratinocytes (HaCaT), as epidermal equivalent, showed flattened morphology like epithelial cells *in vivo*. Nevertheless, the development of two continuously separated cell strata could not be achieved. In parts, MEF cells seemed to overgrow the more slowly proliferating HaCaT cell line. Control frames cultured the whole time under media formed no organotypic skin morphology.

**Figure 9:**
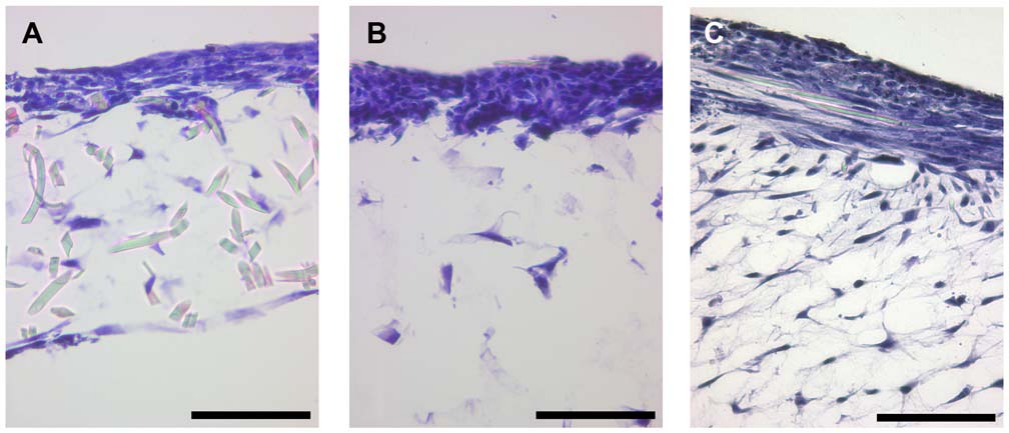
3-dimensional cell culturing. Skin equivalents, cultivated under different medium conditions: Addition of choleratoxin (A), Hydrocortisone (B) or A-2-P (B). bar = 100 µm.

**Figure 10:**
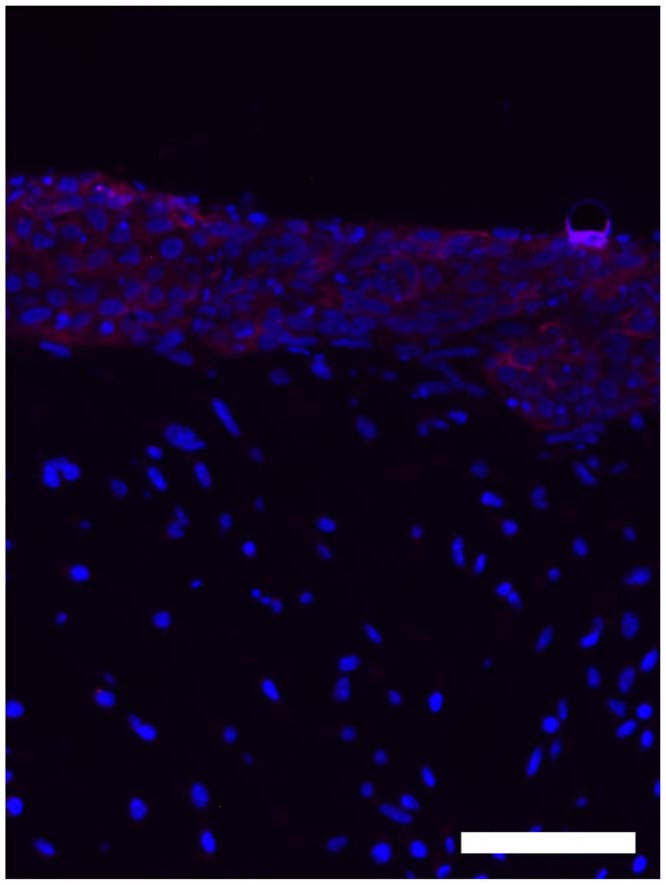
Immunofluorescence microscopy of 3 dimensional, bilayered Cocultures. DAPI staining of cell nuclei in blue, Involucrin as specific marker for HaCaT cells in red. bar = 100 µm.

As a consequence of these results, we wanted to promote keratinocyte growth and reduce the fibroblasts proliferation by changing media conditions. By addition of different supplements to the basal media (DMEM/HAM F12 1∶3) and simultaneous serum reduction from 10% to 1% FCS the formation of the epidermal part could be enhanced (Fig. 9). Frames with accumulated spider dragline silk in the centre cultured for 3 weeks at the air/liquid interface with media containing 50 µg/ml choleratoxin showed bilayered skin morphology. Both epidermis- and dermis-equivalents were clearly separated and continuous (Fig 10). A more decentralized dermis-equivalent resulted from the accumulated spider dragline silk in the center, which provided more adhesion points for fibroblasts. Addition of 50 µg/ml hydrocortisone to the media led to an increase of the epidermal layer in comparison to cultivation with basal media and choleratoxin. Medium supplemented with 0.4 µg/ml A-2-P showed the best results. Fibroblasts as dermal equivalent showed an increase in cell number and formation of extra cellular matrix. Keratinocytes as epidermal equivalent showed a consistent, constantly flat structure. However, the size of the epidermal layer could not be enhanced by A2-P.

The epidermal part of the skin equivalent, the keratinocytes, could be identified specifically by involucrin production of the cells. (Fig. 10)

### Force testing

**Figure 11:**
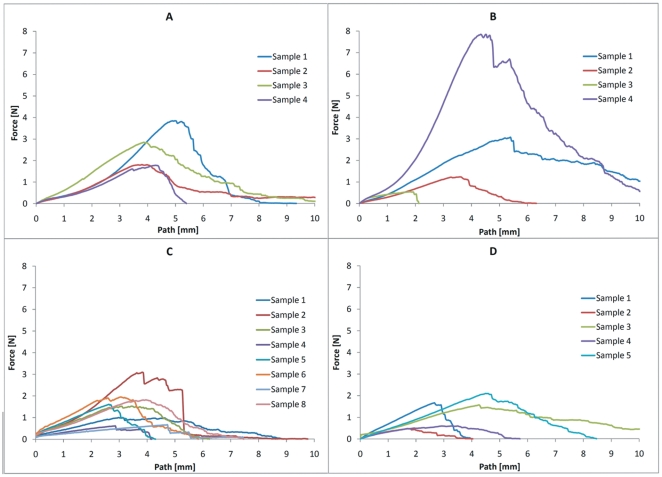
Force testing. The diagrams show graphs describing the generated force of the particular test samples of the control group (n = 5) (A), after a 1 day culture period (n = 4) (B), after a 5 days culture period (n = 8) (C) and after a 21 days culture period (n = 5) (D).

With increasing cultivation time, decreasing mean values of the maximum force were measured. (Fig 11) In each group, wide variations between the individual frames were detected, which caused high standard deviation and so far limited evaluation. (Fig 11 and Table 1) To generate strain-stress curves, the cross sectional area of the constructs must be known. Because of the variability of the different frames, it seemed not to be possible to calculate precisely the cross-sectional area. The cross sectional area consists of two parts, on the one hand the spider silk layers, on the other hand the cell layer. The cross sectional area of the spider silk layer might be calculated by knowing the diameter of one single silk fibre (described in current literature: about 3 µm [21]) and the approximate number of rotations of the spider silk winding machine. But estimating the cross sectional area of the cell layer seemed not to be possible, because the exact cell number during cultivation time is not known. Consequentially, it was not possible to calculate representative stress-values and generate representative stress vs. strain curves. Force-path diagrams pose the adequate alternative for comparison of the different samples within this study.

**Table 1:** Maximum forces.

Maximum tensile strength (MPa)	Control	1 day culture period	5 days culture period	21 days culture period
Mean value	13,28336	15,83733	7,64602	6,39720
Standard deviation	4,37365	16,45962	4,05259	3,54602

The mean value of maximum generated force seems to decrease in all groups over culture time.

## Discussion

In the present study we have demonstrated that spider dragline silk from Nephila spp. can be used as a biological matrix for skin cell culturing. The search and investigation of biocompatible materials, which degrade physiologically, is an important aspect of tissue engineering.

Many groups reported the use of silk biomaterials as possible biomedical applications [31] [32] [33] [34] but none of them used native spider dragline silk as a matrix for skin cell culturing. Spider and silkworm silks differ in many special characteristics: The immunogenic sericin coat of Bombyx mori silk leads to an immune response and has to be removed (degumming process)[35]. Nevertheless, the degummed Bombyx mori silk is well tolerated *in vivo* [36] and for this reason widely used as biomaterial [37]. In contrast to Silkworm silk, native spider silk does not cause such immunogenic response *in vivo* and can be used without any additional processing [25]. To obtain silk from Nephila spp., spiders have to be reared in large rooms, regarding their environments of web construction proportional to their body size [38], they have to be fed with crickets and tended intensively. Today, harvesting of large amounts of spider silk (for industrial standards) is not practicable. Rearing of Bombyx mori silk is less time- and space-consuming but several conditions, like the rearing temperature, have to be considered [39].

Therefore, many groups are working on different strategies for the production of high amounts of recombinant spider silk proteins.

Wen and colleagues reported the creation of a transgenic silkworm, which produced spider silk proteins, combining the chemical and physical properties of spider silk with facilitated harvest of Bombyx mori silk [40]. Alternative strategies included production of spider silk proteins in plants, insect cells or bacteria [41]. Other groups combined spider silk with other materials like poly(D,L-lactide) (PDLLA) [42].

All these strategies implicate some disadvantages. If proteins are produced in cells or bacteria, they first must be harvested and then spun into fibres in complex procedures. But for all that, the biomechanical properties of many newly developed artificial fibres differ from native spider silk fibres. It is hypothesized, that the size of recombinant silk protein might be an important factor irrespective of the mechanical properties of the spun fibre. In this regard it could be shown that when recombinant proteins of similar size like native-sized proteins were produced, the spun fibres exhibited comparable mechanical properties like native dragline silk.[43] In that case, it would be very interesting to acquire more knowledge about the cell culture compatibility of those artificial fibres.

In this study, spider silk was directly isolated from Nephila spp. to benefit from the properties of native dragline silk including cell attachment, elasticity or tensile strength, and unique features of native fibres [44], These factors may be important for promoting the overall strength of a manufactured skin substitute which would be a tremendous advantage in clinical transfer. Mechanical strength of artificial skin is to date an unsolved limiting factor in practical use.

Our studies with woven spider silk fibres at first were impaired by supercontraction of the fibres depending on surrounding conditions. Contact with solvents like water or media result in supercontraction of the fibres up to 50% [45] causing different reaction varying with environmental conditions e.g. high relative humidity (critical point is >70% humidity) [46]. This is an important point for our conducted study regarding the cell culturing procedure and histological embedding. Histological results showed that weaving of the fibres on a stainless steel frame leads to a fixed position. No material artefacts after histological analysis could be observed, but this kind of setup is not applicable for medical use. Further experiments and developments, like an absorbable frame or a knitted spider silk matrix, must be investigated.

An important prerequisite for medical application is the proper storage of spider silk. Storage is possible at normal room humidity (rel. RH) and in the dark preventing UV radiation and succeeding aging of the silk [47].

Results in maximum force testing were wide-ranged. Mean values of the 4 groups seemed to show the tendency of lower generated force after longer culture periods. But due to the high and differing standard deviations, these results are not clearly attributable to the culturing time. (Table 1)

We credit these differences in our results to the variability of native silk and the individual differences of our hand-weaved samples. Nevertheless, these observed variations do not seem to affect the *in vitro* outcome of cell growth on the silk.

After promising results with native silk as matrix for skin substitutes, uniformly producible artificial fibres might facilitate the future for the widespread clinical use of silk-based skin substitutes.

In 1895, von Mangoldt described one of the first methods of epithelial transplantation for skin covering [48]. Currently, medical applications in wound healing are based on these therapeutic backgrounds. Present dermis substitutes can be divided into two groups: temporary and permanent grafts [49]. Temporary models (mostly comprised of synthetic fabrics), must be placed onto the surface and removed after a defined time. These materials are easy to handle and show good results in scar formation. A potential disadvantage could be that these materials must be removed. Permanent materials like Dermagraft [10], Apligraf [50], or full-thickness skin allograft [51] have the advantage that once they are transplanted, permanent wound closure and physiological stability are assured. Disadvantages are the possibility of virus transmission and the limited time period for in-vitro culturing of cells until in vivo transplantation.

Spider silk fibres appear also as being useable as a matrix for permanent wound closure. As it could be shown that spider silk supports the proliferation and guides migration of keratinocytes and fibroblasts, shorter periods of skin substitute preparation may be provided by cell seeding and short culture periods to guarantee stable cell attachment. In this manner, most of the cultivation time would be *in vivo*, but a supporting matrix for cell ingrowth and thus wound closure is supplied. Due to their tensile strength and elasticity, they might contribute to mechanical stability and flexibility of the graft. Earlier studies showed that spider silk fibres also support adhesion and proliferation of other cell types which occur in the dermis of human skin like nerve cells [52], [53] and endothelial cells (data not shown). However, further studies in this set-up should be encouraged as spider silk might turn out to be a close-to-ideal wound coverage.

Taken together, spider silk, woven on stainless steel frames, can be used as matrix for culturing of skin cell lines. Supercontraction of the spider silk proves to be of no consequence by fixation of the fibre to the frame. Not only fibroblast- and keratinocyte-migration and -proliferation can be observed on spider silk, but also coculturing in two continuously separated strata on a matrix consisting purely of spider silk is achieved. Even though the fibroblasts are not embedded into a collagen-gel-matrix (which would provide separated growth of the two cell lines in two layers), a well-balanced bilayered co-cultivation of the two cell lines can be achieved by serum reduction and changing the medium conditions in the described setup. Hereby, disadvantages of collagen-gel matrices like massive contraction can be avoided.

## Materials and Methods

### Breeding of spiders

*Nephila spp.* (first animals were obtained from BTBE; Germany) were bred in our department under animal specific conditions in separate rooms, temperature >17°C and 70% humidity. Male and female spiders were kept together; cocoons of spiders were collected and placed into glass cases to hatch and reach an appropriate size.

### Harvesting of spider dragline silk

Only adult female spiders were used for collection of silk by fixation of the spiders backwards on a polystyrene cube with a gauze bandage and needles. The dragline silk could be pulled out of the spinneret of spiders by stimulation of the *Major ampullate* gland and spun onto a coil or frame with a method previously described [54]. After silk collection, spiders were fed with *Acheta domesticus* and water.

### Frame design, weaving and culturing

Weaving frames were manufactured by a method previously described [27]. Briefly, stainless steel straight wires with a diameter of 0.7 mm (REF 527-070-00, Dentaurum, Ispringen, Germany) were bent to small weaving frames with side lengths from 1–1,5 cm (Fig 1). Frames were placed into a self made, special tuning device (Design and construction by the Institute for Technical Chemistry, University Hannover) for spider silk collection. Spider silk was woven onto the frame in cross patterns to reach a mesh size between 10–100 µm. In this way, two meshes were generated: In the distance of the wires diameter (0.7 mm) one mesh at the upper and one at the lower side of the frame. A spider silk clew was inserted in the 0.7 mm interspace between the upper and lower mesh of the frame. Frames were steam-sterilized at 121°C and a pressure of 2 bar and 100% humidity before use. *In vivo*, skin cells build the body's interface and accordingly have contact to the ambient air from the one side as well as they have to be supplied with nutrients from the other side. To generate these physiological conditions which enhance organotypic cell growth, the constructs have to be cultured at the air liquid interface. Therefore, frames were lifted on a silicone scale which was filled up with polymer fibres (Kisker Biotec). The surface of the medium was below the altitude of the frame. Both were connected by the polymer fibres, which allowed nutrient supply and metabolite evacuation per diffusion (Fig 5).

### Coating of culture plates

To prevent cell attachment on the bottom of cell culture dishes, 1 ml of 0.2% (w/v) Pluronic F-127® in phosphate buffered saline (PBS) (Biochrom AG, Berlin, Germany) was given into a well of a 6-Well-Plate (TPP, Trasadingen, Switzerland) and incubated over night[55]. Solutions were extracted by suction before use.

### Cell culturing

Mouse embryonic fibroblasts (MEF) were cultured in MEF-specific medium containing Dulbecco's modified Eagle's medium (DMEM) High Glucose Cell culture medium (PAA, Pasching, Austria) supplemented with 10% fetal calf serum (FCS) (Biochrom AG), 1% Sodium-pyruvate (PAA) and 1% Gentamycin solution (10.000 µg/ml; Biochrom AG).

Human keratinocytes (Human adult Calcium high temperature keratinocytes, HaCaT) were cultured in HaCaT-specific medium containing DMEM/Hams F12 1∶1 supplemented with 1% FCS, 1% Sodium-pyruvate (PAA) and 1% Gentamycin solution (10.000 µg/ml; Biochrom AG).

For preparing the 3-dimensional skin model, cells from passage 8 to 11 were detached with 1x Trypsine (PAA), collected, counted and centrifuged for 5 minutes at 1200 rpm and resuspended in a equal volume of media.

### 3-dimensional skin model

Frames with cross-woven spider silk were placed into a well and 100 µl cell suspension containing 1×10^6^ MEF in MEF-specific medium was placed onto the middle of the frame. To promote cell attachment on the silk fibres plates were placed into an incubator for 30 minutes, later filled up with 2 ml MEF-specific medium and cultured for two weeks.

On day 15, 1×10^6^ HaCaTs were added on the confluent MEF layer. Before adding HaCaTs, polymer fibres (Kisker biotec) were placed under the frame to achieve regular alignment of the two cell types. Medium was replaced in DMEM/Hams F12 in a 3∶1 ratio supplemented with 1% FCS, 1% Sodium-pyruvate (PAA) and 1% Gentamycin solution (10.000 µg/ml; Biochrome AG, Berlin, Germany). Different experimental settings with modified media conditions were created by adding either 0.4 µg/ml hydrocortisone (Sigma-Aldrich, Bornem, Belgium), 10^−10^ M choleratoxin (Sigma), 50 µg/ml ascorbat-2-phosphat (Sigma) or all of them.

On day 16, cultivation at the air liquid interface like described in Fig. 2 started. Cultivation time at the air liquid interface took additional 3 weeks. Controls were incubated completely under media.

Media were changed three times a week.

### Immunohistology and histology

After culturing time frames were fixed with 4% buffered formaline, dehydrated in a graded series of increasing alcohol concentration, cleared in xylene, embedded in standard procedure in paraffine and cut into 10 µm sections with a microtome (Microm International GmbH, Walldorf, Germany). Slides were deparaffinated, rehydrated by descendent alcohol concentrations and stained with Haematoxyline and Eosine (H&E) or submitted for immunohistological analysis.

For haematoxylin & eosine staining, sections were stained with 1% Haematoxyline (Merck, Darmstadt, Germany) for 5 minutes, rinsed with water for 10 minutes and stained with 2% Eosine (Merck, Darmstadt, Germany) for additional 2 minutes. Slides were dehydrated by ascendant alcohol concentration and mounted in Vectashield® (Vector laboratories, Burlingame, CA, USA).

For immunohistology, sections were permeabilized with 0.1% Triton X-100 in PBS, blocked with PBS containing 2%. FCS. Primary antibodies against involucrine (Abcam, Cambridge, MA, USA) were used in a dilution of 1∶100 in PBS containing 1% FCS., secondary antibodies (Alexa 600, Abcam) were used in a dilution of 1∶200 in the same solvent. Primary antibodies bind to involucrine (Abcam; 1∶100) produced only by keratinocytes. Secondary antibodies identified the primary antibody (Abcam; 1∶200) by emitting a near-infrared fluorescent signal (600 nm). Slides were mounted with Vecta-shield with DAPI (Vectorlaboratorys, Burlingame, CA, USA), which contains 4′,6′-di-amidino-2-phenyl-indol for staining of cell nuclei and analyzed with an inversed fluorescence microscope and AxioVisionH software (both from Zeiss, Jena, Germany).

### LIVE/DEAD Assay

LIVE/DEAD cell viability assay® (Invitrogen, Carlsbad, CA, USA) was done to determine the amount of living cells in the seeded frames. After culturing time, LIVE/DEAD assay was performed following manufacturer's guidelines. Vital cells were able to take up calcein and could be analyzed by green fluorescent light emission (488 nm). Ethidiumbromide homodimer diffuses through the permeable membrane of dead cells and binds into their DNA. Dead cells could be detected by red fluorescent signal (546 nm). LIVE/DEAD assay was analyzed with a fluorescence microscope (Olympus, Hamburg, Germany) by multicolor imaging.

### Force testing

Frames in the size of 3×1 cm were cross-woven with spider silk, seeded with 1×10^6^ MEF and cultured in MEF-specific medium. After culturing periods of 1 (n = 4), 5 (n = 8) and 21 (n = 5) days, the steel-part of the constructs was cut through on two opposite sides, so that the spider silk was spanned between uncoupled pieces of steel frame. Both steel-pieces were clamped in the holder of Instron tension tester (Instron 5565A, Instron Deutschland GmbH, Pfungstadt, Germany) and extended until rupture of the spider silk. The control group (n = 5) was composed of frames, cross-woven with spider silk but left without cells, which were also kept in culture media. Samples were hydrated until such time as the samples were clamped in the machine. The measurements took just some seconds, so that the samples stayed wet during the course of measurement. Because the samples were manually clamped in the machine, a load calibration was done before each measurement. Maximum forces and maximum traverse path were measured and analysed with the Bluehill-2-Software and Microsoft Office Excel 2007.
